# A unique and threatened deep water coral-bivalve biotope new to the Mediterranean Sea offshore the Naples megalopolis

**DOI:** 10.1038/s41598-019-39655-8

**Published:** 2019-03-04

**Authors:** Marco Taviani, Lorenzo Angeletti, Frine Cardone, Paolo Montagna, Roberto Danovaro

**Affiliations:** 1 Institute of Marine Sciences, National Research Council, Via Gobetti 101, 40129 Bologna, Italy; 2 Stazione Zoologica Anton Dohrn, Villa Comunale, 80121 Napoli, Italy; 3 Biology Department, Woods Hole Oceanographic Institution, 266 Woods Hole Road, Woods Hole, MA 02543 USA; 4 Biology Department, University of Bari, Via Orabona 4, 70125 Bari, Italy; 5 Laboratoire des Sciences du Climat et de l’Environnement LSCE/IPSL, CEA-CNRS-UVSQ, Université Paris-Saclay, Avenue de la Terrasse, Île-de-France, Gif-sur-Yvette, 91198 France; 6 Lamont-Doherty Earth Observatory, Columbia University, 61 Route 9W, Palisades, NY 10964 USA; 7 Department of Life and Environmental Sciences, Polytechnic University of Marche, Ancona, Italy, Via Brecce Bianche, 60131 Ancona, Italy

## Abstract

The Gulf of Naples is an example of the most beautiful and biodiverse marine regions of the Mediterranean Sea and of the most impacted areas in terms of industrial activities, large contaminated areas, resource exploitation, infrastructures at sea and maritime transportation. We conducted Remotely Operated Vehicle surveys in the Dohrn Canyon in the Tyrrhenian Sea at approximately 12 NM off Naples metropolitan area, and revealed a hotspot of deep-sea benthic biodiversity of sessile fauna at ca. 400 m depth. The hard bottoms are characterized by a high abundance of charismatic species, such as the habitat forming cold-water corals (CWC) *Madrepora oculata, Lophelia pertusa, Desmophyllum dianthus* in association with the large size bivalves *Acesta excavata* and *Neopycnodonte zibrowii*. This CWC-bivalve co-occurrence represents a novel biotope for the Mediterranean Sea, which coexists with the evidence of severe anthropogenic threats, such as illegal dumping and fishery malpractices that were visually documented during the survey. We recommend the adoption of specific protection measures to preserve these unique deep-sea assemblages showing the uncommon co-existence of such a number of deep-sea species in a single habitat.

## Introduction

In the 19^th^ century, the beauty and biological richness of the Gulf of Naples (GoN) was such to convince Anton Dohrn to found there in 1872 the first marine station in the world to demonstrate the validity of the Darwin theories on evolution using marine organisms. The following 150 years have witnessed the unbridled growth of scientific studies in the gulf, along with the explosion of the industrial and urban development of the city of Naples and adjacent coast, especially after World War II^1^, which determined a progressive increase of the anthropogenic pressures. Today, the Naples metropolitan area accounts for ca. 4.5 million inhabitants and the entire population of the gulf is distributed along a mere 12 km coastal stretch. The presence of illegal dumping and untreated sewage, along with the presence of wide contaminated areas, intense maritime transportation, infrastructure at sea, other direct and indirect anthropogenic stressors^2–13^ have progressively threatened the coastal ecosystems of this gulf, determining also a progressive loss of marine habitats.

**Figure 1:**
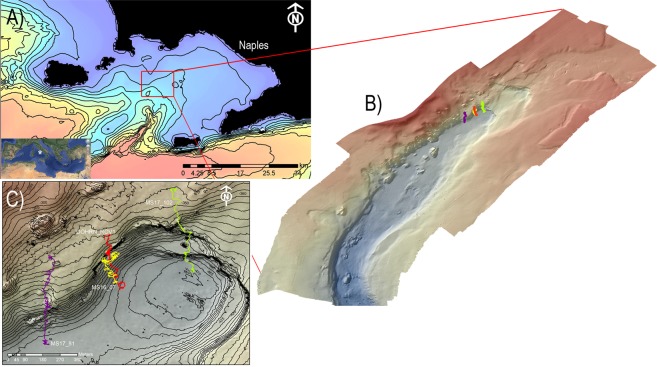
(**A**) Location map of the Gulf of Naples (white star in the inset. Basemap data: Google, SIO, NOAA, US Navy, NGA, GEBCO; Image Landsat/Copernicus) showing the three main canyons dissecting the Campanian shelf. Bathymetry from^52^ (http://www.emodnet-bathymetry.eu/), contour lines spacing 100 m; (**B**) detail of Dohrn Canyon (red square in **A**) and location of the four ROV dives performed in the area. (**C**) ROV tracks plotted over the morphobathymetric map. Note the sector of the canyon wall between 350 and 470 m depth where deep-water corals and large bivalves do occur. Contour line spacing 5 m. Maps were created using ArcGIS software by Esri (www.arcgis.com). ArcGIS and ArcMap are the intellectual property of Esri. Morphobathymetry were generated using CARIS HIPS and SIPS release 9 software (http://www.caris.com).

Despite a long tradition of biological studies that renders the GoN one of the most intensively investigated marine areas of the world, little is known about the ecology and environmental status of the deep-sea habitats of the gulf, whose hard bottom has been so far investigated only in terms of geological setting. A prominent submarine feature of the gulf is the Dohrn Canyon, a bifurcate structure (Fig. 1) that indents perpendicular to the coastline the continental shelf 12 NM from the Naples megalopolis, beginning at ca. −250 m and sharply declining down to ca 1300 m in the Tyrrhenian plain^14,15^. This inactive canyon, part of the Magnaghi-Dohrn canyon system^16^ and consisting of a western and an eastern branch, is thought to have been formed in response to relative sea-level fall during the last glaciation enhanced by local subsidence, cutting through Pleistocene sediments and the Campana Ignimbrite^14^. The water mass structure of the GoN is predominantly linked to the main circulation of the southern and mid-Tyrrhenian Sea, with influences from local factors, such as the wind stress and the river runoff. The two main water masses flowing in the GoN are the Modified Atlantic Water (MAW) that occupies the upper 50–100 m and the Levantine Intermediate Water (LIW) located below ~200–300 m, with salinity of 38.65, temperature of 14.2 °C and a potential density (σ_θ_) of 29.0 kg m^−3^ ^17–19^. Depending on the season, other water masses can be recognized within the GoN, such as the Tyrrhenian Intermediate Water (TIW) formed during winter mixing at depths down to ~150 m and the Tyrrhenian Surface Water (TSW) found above 75 m as the result of summer warming and freshening of the TIW^20^.

In 2016, the first ecological exploration of the deep-sea habitats of the Dohrn Canyon was launched by means of Remotely Operated Vehicle (ROV) focusing on the steep walls of the northern flank of its western branch (Fig. 1B,C). The survey unveiled for the first time the presence of charismatic benthic life in the canyon system, with biological traits previously unreported for the entire Mediterranean basin.

## Results

### The living community

**Figure 2:**
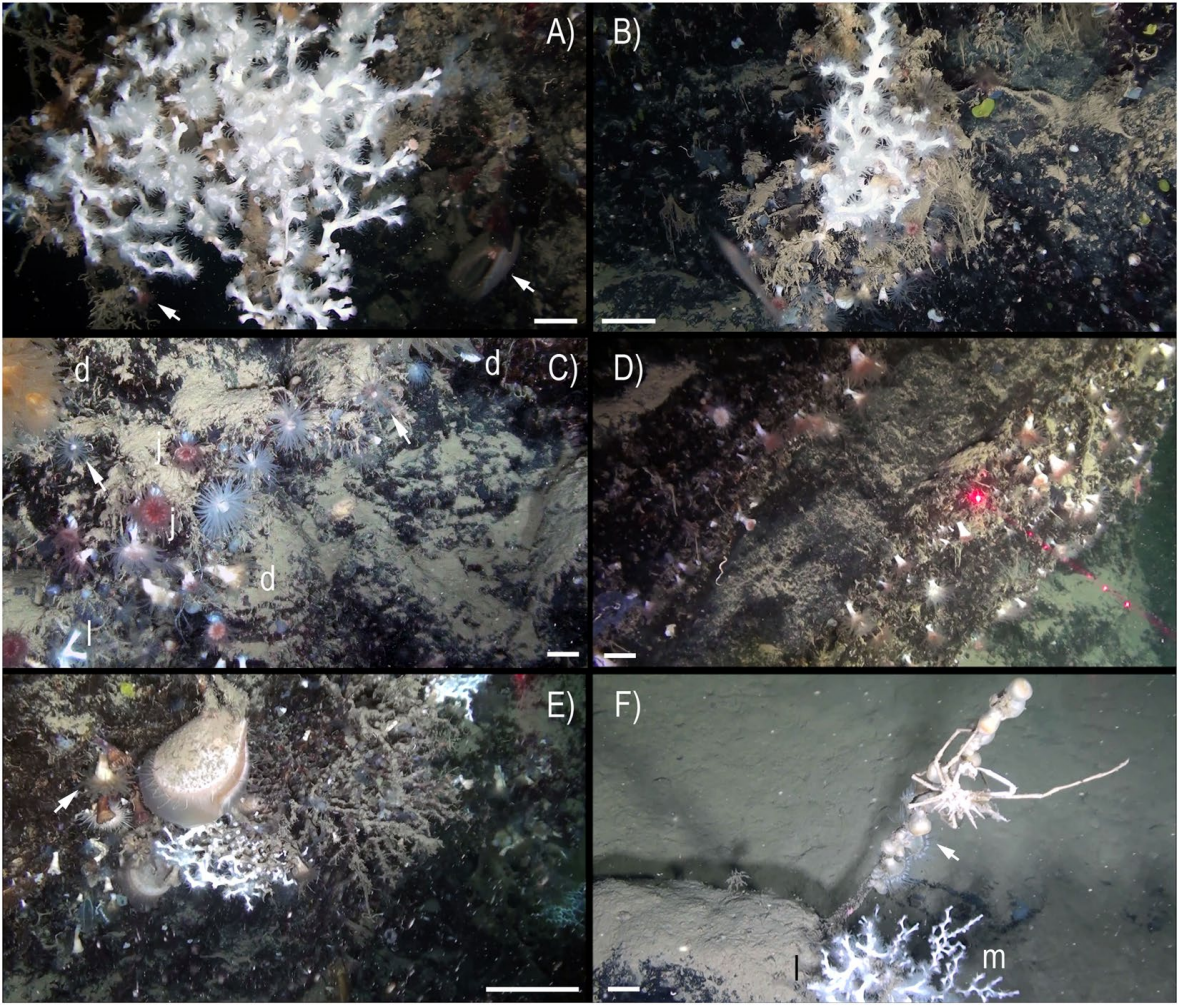
Main frame coral builders and most noticeable fauna in the Dohrn Canyon: (**A**) the colonial scleractinian *Lophelia pertusa* with expanded polyps; arrows indicate *Acesta excavata* on the right foreground and the solitary coral *Javania cailleti* on the left foreground (st. MS16_7, −386 m) bar = 2 cm; (**B**) live colony of the scleractinian *Madrepora oculata* (st. MS16_7, −391 m, bar = 2 cm); (**C**) solitary coral community, with adult and juvenile *Desmophyllum dianthus* (d), *J. cailleti* (j), the sea anemone *Protanthea simplex* (arrows), plus on the left side a juvenile *L. pertusa* (l) (st. MS16_7, −385 m) bar = 1 cm; (**D**) curtains of solitary corals (*Desmophyllum dianthus*, *Javania cailleti*) rimming the layered bedrock (st. DOHRN_ROV_1, −385 m) bar = 1 cm; (**E**) the large limid *A. excavata* among solitary (*D. dianthus*: white arrow) and colonial (*M. oculata*) scleractinian corals, both alive and dead (st. MS16_7, −415 m) bar = 10 cm; (**F**) mud-draped substrate with small colonies of *M. oculata* (m) and *L. pertusa* (l), arrow indicates a group of small anemone (*Amphianthus dohrnii*) above the decapod *Anamathia rissoana* crawling on a undefined stalk (st. MS16_7, −412 m) bar = 1 cm.

**Figure 3:**
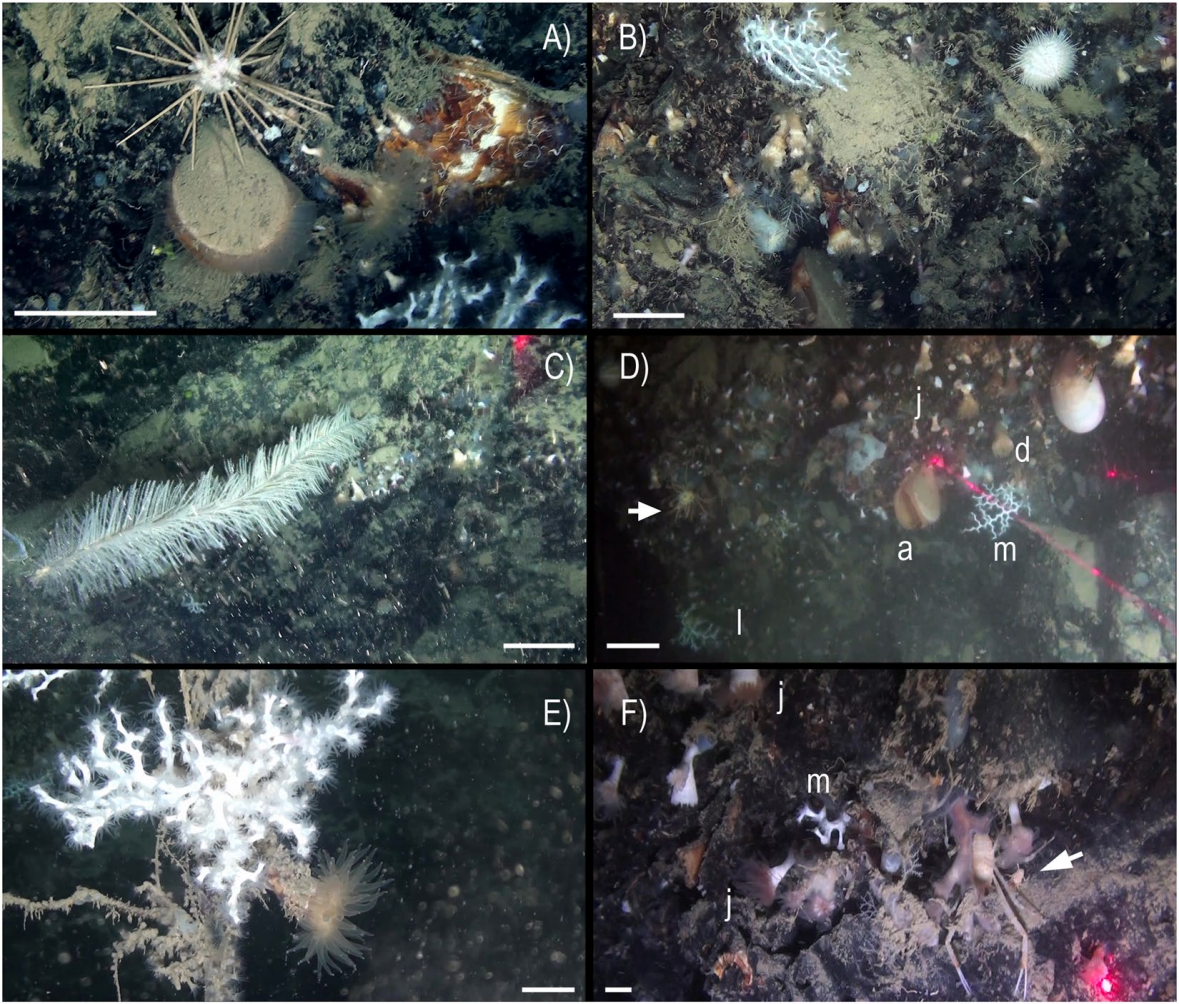
Details of the fauna inhabiting the Dohrn Canyon: (**A**) the sea-urchin *Cidaris cidaris* crawling next to a live *Acesta excavata*, note a living *Neopycnodonte zibrowii* to the right, plus various solitary corals (*Desmophyllum dianthus*) and *Madrepora oculata* in the background (st. MS16_7, −386 m) bar = 5 cm; (**B**) live and dead *M. oculata* colonies, *D. dianthus*, *A. excavata*, and the echinoid *Gracilechinus* cf. *acutus* (st. MS16_7, −384 m) bar = 5 cm; (**C**) the antipatharian *Parantipathes larix* (st. MS16_7, −406 m) bar = 5 cm; (**D**) image documenting the taxonomic complexity of the sessile fauna in the canyon: *A. excavata* (a), *M. oculata* (m), *L. pertusa* (l), *D. dianthus* (d) and *J. cailleti* (j), arrow points the decapod *Anamathia rissoana* (st. DOHRN_ROV_1, −377 m) bar = 10 cm; (**E**) the solitary coral *D. dianthus* with fully expanded polyp next to a colony of *L. pertusa*, note also the colonial sea anemone *Epizoanthus* sp. colonizing a lost longline (st. MS16_7, −391 m) bar = 5 cm; (**F**) coral assemblage dominated by *J. cailleti* (j), small colony of *M. oculata* (m) and the decapod *Munida intermedia* sheltered among corals, arrow indicates juvenile specimens of *M. intermedia* (st. DOHRN_ROV_1, −384 m) bar = 2 cm.

**Figure 4:**
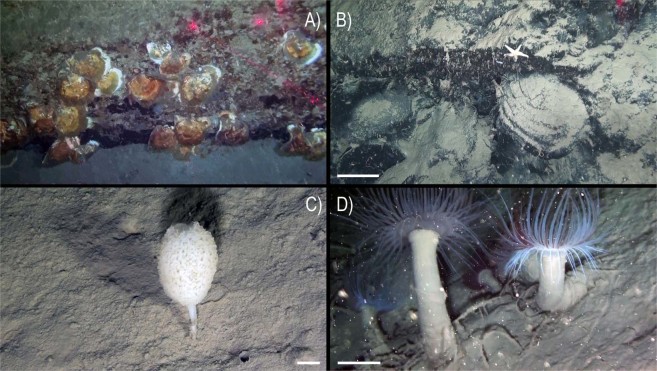
Dohrn Canyon: (**A**) *Neopycnodonte zibrowii* string settling on the emerging layered bedrock (st. MS17_102) −446 m; (**B**) mud-draped bedrock with dead and blackened cemented shells of *N*. *zibrowii*, note an undetermined Ophidiasteridae seastar on top (st. MS16_7, −388 m) bar = 10 cm; (**C**) the sponge *Stylocordyla pellita* stemming from the canyon’s muddy bottom (st. MS16_7, −435 m) bar = 1 cm; (**D**) dense aggregation of *Pachycerianthus* cf. *dohrni*, note longline trapped among the cnidarians (st. MS16_7, −411 m) bar = 5 cm.

**Figure 5:**
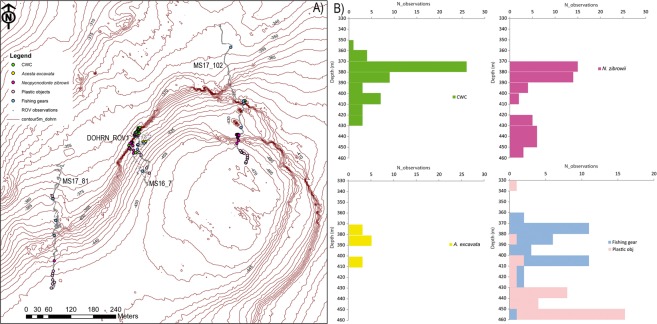
(**A**) Habitat map based upon four ROV surveys carried out in the western branch of the Dohrn Canyon with respect to the most relevant biological taxa and marine litter observed there; it documents a widespread distribution of *Neopycnodonte zibrowii* in the canyon head at steeper loci at the sites DOHRN-ROV_1/MS16_7 and MS17_102 but only sporadic at MS17_81; co-occurring CWC presence (*Lophelia pertusa*, *Madrepora oculata*, *Desmophyllum dianthus*) is less obvious loci at the site MS17_102; *Acesta excavata* is relatively frequent at the sites DOHRN-ROV_1/MS16_7. (**B**) Frequency of organisms and litter (x-axis) with respect to the depth range (y-axis); it documents a higher biodiversity between 370 and 390 m water depth; the stronger impact by fishing gears is located between 370 and 410 m, associated with hard substrates, whereas the maximum dumping impact is found between 430 and 460 m on muddy bottoms.

**Figure 6:**
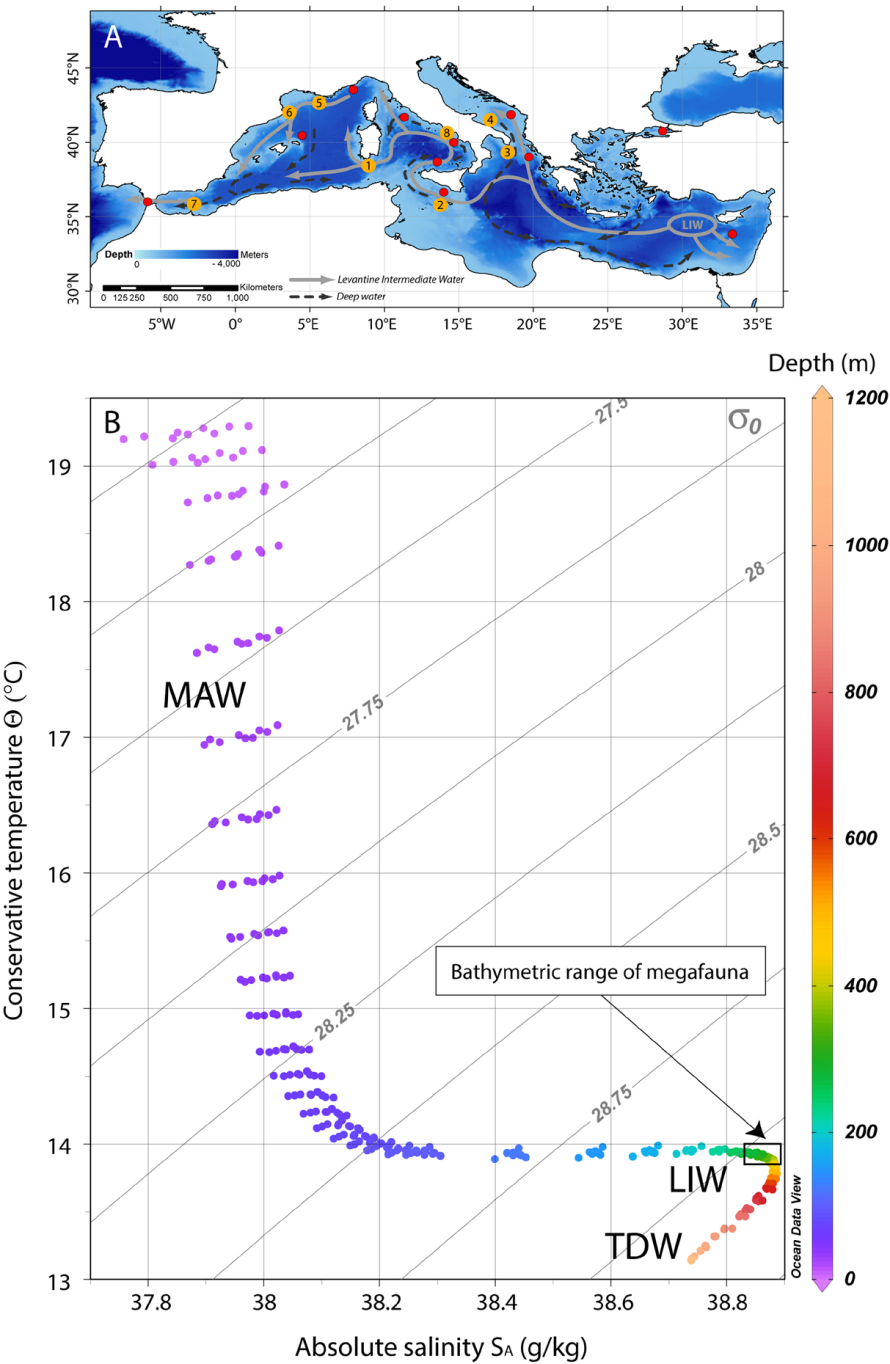
(**A**) Map of the Mediterranean Sea showing the main path of the LIW and the deep water currents (modified from)^53,54^; the major cold-water coral Provinces are reported^27^, upgraded to including the new Dohrn Canyon site: 1. South Sardinia, 2. South Malta, 3. Santa Maria di Leuca, 4. Bari Canyon, 5–6. Gulf of Lions, 7. Melilla, 8. Dohrn Canyon. (Map modified from)^24^. (**B**) T-S plot of 8 different depth profiles sourced from the World Ocean Atlas 2013 (WOA13) database^55,56^ in the area of the Gulf of Naples (area between 40°52′30″N and 40°22′30″N; 13°52′30″E and 14°52′30″E). Isopycnals are calculated at 0 m (σ_0_). MAW: Modified Atlantic Water, LIW: Levantine Intermediate Water, TDW: Tyrrhenian Deep Water. The plot was created using Ocean Data View (Schlitzer, R., Ocean Data View, http://odw.awi.de, 2015).

An abundant and diverse sessile fauna was observed by ROV image analysis (Figs 2–4). Several macro-invertebrates species were concentrated on rocky bottoms at depths of 350–470 m (Fig. 5), corresponding to the depth of the LIW (Fig. 6). We report here the presence of the frame-building cold-water colonial scleractinian corals *Lophelia pertusa* and *Madrepora oculata*. These species are key ecosystem engineers in deep-sea habitats of the Atlantic Ocean and have been reported with a fragmented distribution in the Mediterranean Sea^21,22^ but were never reported previously in the GoN. Differently from what observed so far in the Mediterranean Sea, where cold-water coral habitats are predominantly characterized by *M. oculata*, in the Dohrn Canyon *L. pertusa* is the most abundant species, especially on the northwestern flank where as many as 10 colonies of each species have been counted on a distance of 235 m between 375–455 m water depth. Our ROV images and videos indicate that both species occur as small interspersed colonies on the rocky substrate (Figs 2A,B,E and 3B,E), isolated or admixed (Fig. 2F), and with polyps fully expanded (Figs 2A,B,F and 3B,E). On the canyon walls, we also found conspicuous populations of solitary corals (Figs 2C–E and 3B,D,F), including *Javania cailleti* and *Desmophyllum dianthus* (a target species for paleoclimatically-oriented studies^23^). Such solitary corals settle on bedrock overhangs forming dense aggregations of up to more than 700 ind. m^−2^, with the tentacles facing down, and often unevenly rimming the layered substrate (Fig. 2D). The sea anemone *Protanthea simplex* is equally abundant (Fig. 2C), often associated with solitary corals or settled on the dead frames of *M. oculata* and *L. pertusa*. This piezotolerant species, spanning from infralittoral to bathyal depths in the northern Atlantic, has been previously reported only once in the Mediterranean, in the Strait of Sicily^24^. Another unique finding is the discovery of the presence of large bivalves in association with such CWC (Fig. 5; Table 1), namely the limid *Acesta excavata* (Figs 2E and 3A,D), and the giant deep-sea oyster *Neopycnodonte zibrowii* (Fig. 4A), which colonize the vertical cliff of the canyon. These large bivalves are frequently reported in the Atlantic Ocean, but seldom documented alive in the Mediterranean Sea^25–27^, where their presence has been so far documented only in the western basin or as Pleistocene fossil in the eastern basin. Individuals attain a size of ca. 20 cm that is comparable with the NE Atlantic populations^28^. Individuals of *A. excavata* have been observed byssate on the wall at 380 m water depth down to the basal part of the canyon flank at 415 m. The long-lived oyster *N. zibrowii* is recorded in all ROV transects along the Dohrn Canyon (Figs 3A and 4A) but with variable density, occurring as individuals or aggregations (strings) in a depth range of 375–415 m. Remarkably, clusters of dead and black-coated *N. zibrowii* occur cemented at the canyon’s wall, some of which fouled by living epifauna that includes CWC. Overall we counted more than 120 living *N. zibrowii* reaching a maximum density of ca. 20 ind. m^−2^ in the northwestern part of the canyon between 375–395 m (Fig. 4B). The canyon’s wall is inhabited by a number of other mega- and macrobenthic taxa including further cnidarians, sponges, polychaetes, molluscs, bryozoans, echinoderms, crustaceans, adding to the total biodiversity (see species list in Table 2). Other cnidarians include rare specimens of the antipatharian *Parantipathes larix* (Fig. 3C), the sea fan anemone *Amphianthus dohrnii* (Fig. 2F) abundant on long lines entangled in the wall, and an unidentified species of *Epizoanthus* sp. (Fig. 3E). The sponge assemblages are dominated by the encrusting species *Desmacella inornata* and *Hexadella* sp., and by a few massive and tubular sponges (i.a., *Spongosorites flavens*). Serpulid polychaetes, encrusting and erect bryozoans, byssate (*Asperarca nodulosa, Delectopecten vitreus*) and cemented (*Spondylus gussonii*) bivalves contribute to the high biodiversity of hard substrates of the canyon. The investigated system hosts also some vagrant macro-invertebrate species, such as the pencil urchin *Cidaris cidaris* (Fig. 3A), *Gracilechinus* cf*. acutus* (Fig. 3B), the asteroid *Astropecten* sp., and the decapods *Munida intermedia* (Fig. 3F) and *Anamathia rissoana* (Fig. 2F). The colonial coral framework provides a cryptic habitat populated by a diversified fauna such as the pink fish *Bellottia apoda* and unidentified crustaceans.

**Table 1:** Number of organisms identified through ROV analysis for each transect and length of the hard and mobile substrates.

	DOHRN_ROV01	MS16_7	MS17_81	MS17_102	N_tot	Tot_density (rec. m^−2^)
Recovery						N_rec. m^−2^
*N_zibrowii*	17	2	0	108	127	0.052
*A_excavata*	7	3	0	0	10	0.004
CWCs	45	36	0	8	89	0.037
	(m)	(m)	(m)	(m)	**Total length (m)**	Total area (m^2^)
Mobile substrate	198.95	385.20	806.47	504.97	**1895.58**	5686.74
Hard substrate	235.09	423.49	33.48	115.72	**807.77**	2423.31
Mobile + Hard substrate	434.04	808.69	839.95	620.68	**2703.36**	8110.08

The distribution density of the organisms, reported as the number of individuals per unit area, refer only to the hard substrate.

**Table 2:** Taxa identified on the canyon rocky wall and adjacent muddy bottoms, based upon ROV records.

Phylum	Class	Order	Family	Species	WALL	BOTTOM
Porifera	Hexactinellida	Lyssacinosida	Leucopsacidae	*Oopsacas minuta* Topsent, 1927	x	
	Demospongiae	Tetractinellida	Ancorinidae	*Jaspis incrustans* (Topsent, 1890)	x	
				*Holoxea furtiva* Topsent, 1892	x	
		Suberitida	Stylocordylidae	*Stylocordyla pellita* (Topsent, 1904)		x
			Halichondriidae	*Spongosorites flavens* Pulitzer-Finali, 1983	x	
		Axinellida	Raspailiidae	*Raspaciona calva* Sarà, 1958	x	
				*Eurypon hispidulum* (Topsent, 1904)	x	
		Poecilosclerida	Hymedesmiidae	*Phorbas fictitius* (Bowerbank, 1866)	x	
			Latrunculiidae	*Sceptrella insignis* (Topsent, 1890)	x	
		Desmacellida	Desmacellidae	*Desmacella inornata* (Bowerbank, 1866)	x	
		Agelasida	Hymerhabdiidae	*Prosuberites longispinus* Topsent, 1893	x	
				*Hymerhabdia intermedia* Sarà & Siribelli, 1960	x	
		Verongiida	Ianthellidae	*Hexadella* sp.	x	
	Demospongiae			sp. 1	x	
				sp. 2	x	
Cnidaria	Anthozoa	Antipatharia	Schizopathidae	*Parantipathes larix* (Esper, 1788)	x	
		Penicillaria	Arachnactidae	sp.		x
		Spirularia	Cerianthidae	*Pachycerianthus* cf. *dohrni* (Van Beneden, 1923)		x
		Actiniaria	Gonactiniidae	*Protanthea simplex* Carlgren, 1891	x	
			Hormathiidae	*Amphianthus dohrnii* (Koch, 1878)	x	
				cf. *Calliactis parasitica*		x
			Sagartiidae	*Kadophellia bathyalis* Tur, 1991	x	
		Scleractinia	Oculinidae	*Madrepora oculata* Linnaeus, 1758	x	
			Caryophylliidae	*Caryophyllia calveri* Duncan, 1873	x	
				*Desmophyllum dianthus* (Esper, 1794)	x	
				*Lophelia pertusa* (Linnaeus, 1758)	x	
			Flabellidae	*Javania cailleti* (Duchassaing & Michelotti, 1864)	x	
			Stenocyathidae	*Stenocyathus vermiformis* (Pourtalès, 1868)	x	
		Zoantharia	Epizoanthidae	*Epizoanthus* sp.		x
Mollusca	Bivalvia	Pectinida	Pectinidae	*Delectopecten vitreus* (Gmelin, 1791)	x	
			Spondylidae	*Spondylus gussonii* O. G. Costa, 1830	x	
		Limida	Limidae	*Acesta excavata* (Fabricius, 1779)	x	
		Ostreida	Gryphaeidae	*Neopycnodonte zibrowii* Gofas, Salas & Taviani, 2009	x	
		Arcida	Arcidae	*Asperarca nodulosa* (O. F. Müller, 1776)	x	
Annelida	Polychaeta	Eunicida	Eunicidae	*Eunice norvegica* (Linnaeus, 1767)	x	
		Sabellida	Serpulidae	*Serpula vermicularis* Linnaeus, 1767	x	
				*Bathyvermilia eliasoni* (Zibrowius, 1970)	x	
				sp. 1	x	
				sp. 2	x	
				sp. 3	x	
		Echiuroidea	Bonelliidae	*Bonellia viridis* Rolando, 1821	x	
Arthropoda	Malacostraca	Euphasiacea			x	x
		Decapoda	Munididae	*Munida intermedia* A. Milne Edwards & Bouvier, 1899	x	
			Epialtidae	*Anamathia rissoana* (Roux, 1828)	x	
			Paguridae			x
Bryozoa	Stenolaemata			sp.	x	
	Gymnolaemata			sp. 1	x	
				sp. 2	x	
				sp. 3	x	
				sp. 4	x	
Chaetognatha	Sagittoidea	Aphragmophora	Sagittidae	*Sagitta* spp.	x	
Echinodermata	Asteroidea	Paxillosida	Luidiidae	*Luidia ciliaris* (Philippi, 1837)	x	
			Astropectinidae	*Astropecten* sp.		x
		Valvatida	Ophidiasteridae	sp.	x	
	Echinoidea	Cidaroida	Cidaridae	*Cidaris cidaris* (Linnaeus, 1758)	x	
		Camarodonta	Echinidae	*Gracilechinus* cf. *acutus* (Lamarck, 1816)	x	
Chordata	Actinopterygii	Anguilliformes	Ophichthidae	*Nettastoma melanurum* Rafinesque, 1810		x
		Myctophiformes	Myctophidae		x	x
		Gadiformes	Phycidae	*Phycis blennoides* (Linnaeus, 1766)		x
			Macrouridae	*Coelorinchus caelorhincus* (Risso, 1810)		x
		Ophidiiformes	Bythitidae	*Bellottia apoda* Giglioli, 1883	x	
			Trachichthyidae	*Hoplostethus mediterraneus* Cuvier, 1829	x	
		Perciformes	Sparidae	*Pagellus bogaraveo* (Brünnich, 1768)	x	
			Trichiuridae	*Lepidopus caudatus* (Euphrasen, 1788)	x	

Nekton diversity during the ROV survey revealed the presence of a limited number of species. Shoals of *Pagellus bogaraveo* were observed swimming next to the canyon wall, and the silver scabbardfish *Lepidopus caudatus* was abundant near the wall and the bottom of the canyon. ROV explorations were extended also on the adjacent muddy bottoms of the canyon. Here we observed the presence of sparse cerianthiids, unidentified Paguridae, other benthopelagic fishes and sponges (Fig. 4C). In particular, the Ceriantharia *Pachycerianthus* cf. *dohrni* reached here densities up to 5–6 ind. m^−2^ (Fig. 4D). Finally, the ROV images documented a high zooplankton biomass, dominated by Euphausiacea and Chaetognata.

### Dating the Canyon occupancy by the coral-bivalve biotope

**Figure 7:**
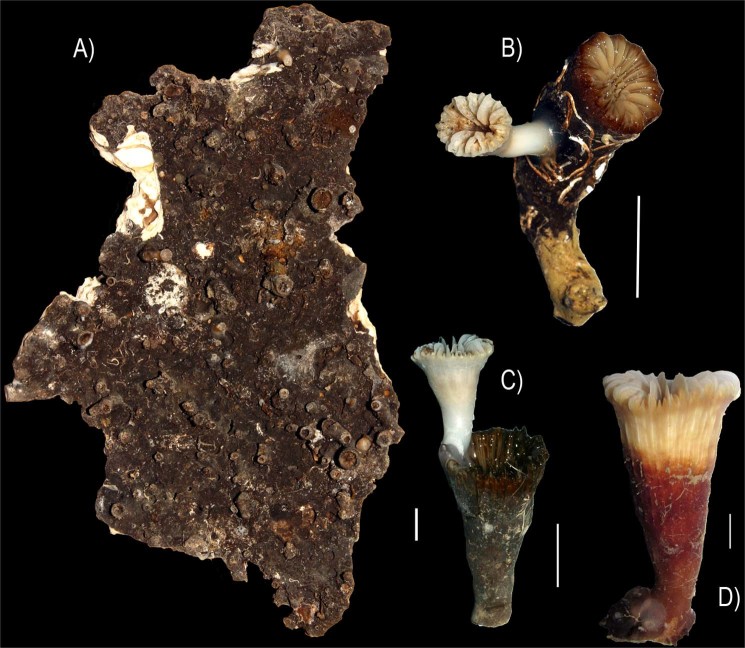
Samples taken by the ROV robotic arm at st. DOHRN_ROV_1: (**A**) dead and partly broken shell of *Neopycnodonte zibrowii* that provided a calibrated age of 5496 yr cal BP; note the black oxide patina which also affects numerous fouling organisms as solitary corals and serpulid polychaetes (−373 m); (**B**) a juvenile live-collected *Javania cailleti* on the side of a dead and patinated *Caryophyllia calveri* coral (−372 m); (**C**) juvenile live-collected *Desmophyllum dianthus* growing on the calyx of an older specimen (−371 m); (**D**) this *Desmophyllium dianthus* was collected alive, but its skeleton was already stained by an incipient oxide patina (−377 m). Scale Bars = 1 cm.

We have observed the common presence of subfossil oysters cemented to the wall (Fig. 4A,B). Some dead *D. dianthus* and *Caryophyllia calveri* appear also completely patinated by ferromanganese oxides, serving as attachment bases for living individuals of *D. dianthus* and *J. cailleti*, respectively (Fig. 7B,C). Noticeably, incipient oxide patination was observed on some live *D. dianthus* corals (Fig. 7D), although such polymetallic patina often testifies at a prolonged *post-mortem* exposure of the calcareous skeleton within the water column^29,30^. One dead valve of *N. zibrowii* coated by a thick black ferromanganese patina (Fig. 7A) was collected from the wall and radiocarbon dated at the Poznan Radiocarbon Laboratory in Poland, providing a calibrated age of 5496 yr cal BP (2σ range: 5329–5588) (Table 3). We hypothesize that this age might represent the time of coral-bivalve settlement of the Dohrn Canyon. The date matches well with the timing of re-colonization that followed the collapse of CWC-growth in the eastern Mediterranean Sea due to oxygen deficiency in intermediate and deep waters between 10.8–6.1 kyr BP^31^.

**Table 3:** Radiocarbon data from the Poznan Radiocarbon Laboratory, Poland.

Laboratory Code#	Species	Radiocarbon age (^14^C-yr BP)	Calibrated age Median probability (yr cal BP)	Calibrated age 2σ range (yr cal BP)
Poz-99025	*Neopycnodonte zibrowii*	5180 ± 35	5496	5329–5588

The ^14^C age was converted into calendar years (cal. yr BP, BP = AD 1950) by using the Marine13^50^ calibration curve and the Calib7.0.4 program^51^. Prior to calibration, the age was corrected for an extra 49 ± 42 years regional reservoir effect (ΔR) using the value reported in the Marine Reservoir Correction Database (http://calib.qub.ac.uk/marine/). The calibrated age range reflects the 95.4% probability window (2σ).

Thus, to the best of our knowledge, the Dohrn Canyon became home of this peculiar biotope since the mid Holocene at least, along with the onset of the LIW at the end of the sapropel S1 event at 6.1 kyr BP. We have no indication of a previous presence of CWC in this site, although it cannot be excluded *a-priori* given the Late Pleistocene *L. pertusa* recorded from off the Gulf of Naples^32^.

### Anthropogenic impact

**Figure 8:**
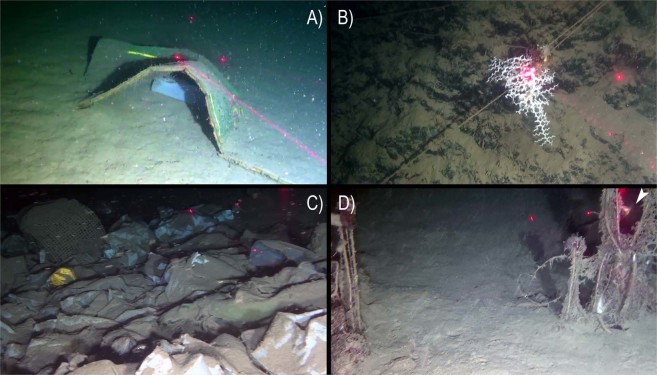
Typology of macroscopic anthropogenic impacts in the Dohrn Canyon including litter and lost fishing gears: (**A**) dumped mattress and plastic bag (st. ROV_DOHRN_1, −430 m); (**B**) colony of *Madrepora oculata* grown on lost longline (st. ROV_DOHRN_1, −375 m); (**C**) accumulation of dumped bags and other plastic objects completely covering the bottom (st. MS17-102, −453 m); (**D**) ghost net on a relatively bare substrate, note the solitary coral *Desmophyllum dianthus* (arrow) sitting on the same net (st. MS17_102, −453 m).

The ROV survey provided evidence of severe anthropogenic impacts on the Dohrn Canyon (Figs 5 and 8). Dohrn_01, MS17-81 and MS17_102 ROV tracks on the canyon’s bottom documented the abundant presence of marine litter even of large size (Fig. 8A,C), as well as lost nets and longlines seen enveloping also epifaunal megabenthos. A high number of longlines was reported entangled on substrate asperities (Fig. 8B,D) often close to colony of cold-water corals and bivalves. Longlines served also as attachment substrate for cnidarians, including *M. oculata* and *D. dianthus* (Fig. 8B,D). Our data pointed out the presence on an impressive amount of illegal dumping of garbage bags, so intense to completely drape the sea-bottom at places (Fig. 8E). Marine litter has been classified following the MSFD guidance provided by^33^, with density indicated as items/100 m based on the total length of the ROV tracks (Fig. 5B). Marine litter attains an overall density of 5.03 items/100 m, considering a total of 136 different objects (93 plastic objects: 3.44 items/100 m; 43 lost fishing gears: 1.59 items/100 m). Plastic objects are computed only for muddy bottoms (4.91 items/100 m), whereas lost fishing gears (5.32 items/100 m) are evident on hard substrates (Figs 5 and 8B,D; Table 4).

**Table 4:** Number of litter identified through ROV analysis.

Recovery	DOHRN_ROV01	MS16_7	MS17_81	MS17_102	N_tot	Density (N_rec/Tot_lenght)	Density (N_tot_rec/100 m)
						N_rec/100 m	N_tot_rec/100 m
Plastic objects (A1, A3, A5)	6	0	10	77	93	3.44*	5.03
Fishing objects (C6)	20	10	6	7	43	1.59**	

Density of litter is reported as number of records for a distance of 100 m as indicated by the Guidance on Monitoring of Marine Litter for the Mediterranean MSFD^33^. The total density refers to the density of the identified objects for the entire length of the four ROV under study; Density refers to the density of the objects identified in mobile sediments (*) and hard substrates (**).

## Discussion

Despite the fact that intensive biological investigations have been conducted in the GoN since the foundation of the Stazione Zoologica in 1872, information on deep-sea benthos has remained surprisingly very scant and limited to mobile bottoms^34–36^. The hard bottoms of the Dohrn Canyon reveal further elements of seafloor heterogeneity in the Gulf, which increase the incredibly rich topographic features of the coastal habitats, with all predictable ecological consequences^37^. It is surprising, however, that the presence of such a highly diverse habitat was so close to the shoreline and remained unnoticed until present. This finding further strengthens the evidence of the still insufficient knowledge of the deep-sea realm even in proximity of the coast^38,39^, and of the deep-sea Mediterranean biodiversity in particular^40,41^.

With respect to the cold-water coral habitats of the Mediterranean basin, the Dohrn Canyon is of particular oceanographic and ecological interest as it represents an element of potential connectivity with other deep-water coral populations of the basin. The GoN indeed is peripheral, but still on the track of the LIW (Fig. 6A), which is hypothesized to control the distribution of major deep-water coral grounds in the Mediterranean basin^42^.

The deep-sea assemblages revealed by this study indicate that the Dohrn Canyon is a highly diverse cold-water coral site of the eastern Tyrrhenian Sea, for which available information previously confirmed the occurrence of living specimens of *M. oculata*, *L. pertusa* and *D. dianthus* (pers. unpublished data), but never the contextual presence of *N. zibrowii* and *A. excavata* except in the case of subfossil Pleistocene records^30,43^.

A unique trait of the faunal assemblage of the Dohrn Canyon, however, is the tight co-existence of living specimens of the bivalves *A. excavata* and *N. zibrowii* with the cold-water corals, which has never been previously reported in the Mediterranean Basin. The assemblages of the Dohrn Canyon share several common traits with those inhabiting the Whittard Canyon of the eastern Atlantic Ocean^44–46^. Both canyons share, indeed, the presence of the same large bivalves of *A. excavata* and *N. zibrowii*, although with a much higher bivalve density in the Whittard than in the Dohrn Canyon. However, in both areas *N. zibrowii* appears to preferentially settle at slightly deeper depths than *A. excavata*. Conversely to what reported from the Whittard Canyon, solitary corals predominate in the Dohrn Canyon.

The new finding reported here from the Dohrn Canyon clearly indicates that the GoN is a hot spot of deep-sea benthic diversity coexisting with high levels of anthropogenic impact. The canyon head, indeed, is very close to the Naples megalopolis, and is subjected to a severe dumping and ghost- fishing impact, that were clearly documented by ROV images. Data on the number of large litter, and plastic debris, and of fishing nets and lines in the deep are still very scant^47,48^, so that remains difficult to provide sound comparisons. However, we did not find evidence of trawling and topographic elements of physical damage and habitat destruction (e.g., dredging). Data reported in the present study, thus suggest that hot spot of deep-sea benthic biodiversity can coexist with these impacts (litter, long lines) if the latter are not coupled with the habitat destruction. We call for the preservation of these unique deep-sea habitats by adopting protection measures from bottom contact fishing and other sources of impact. This goal could be partially achieved by granting to the Dohrn Canyon the status of Site of Community Importance (SCI) within the European Commission Habitats Directive, and by starting action to establish a deep-sea Marine Protected Area in this Mediterranean area.

## Methods

A new swath bathymetry on a sector of Dohrn Canyon was acquired during the second cruise using Kongsberg Simrad 8160 Multibeam Echosounder hull-mounted with a frequency of 44 kHz. All data were plotted in the Transverse Mercator – UTM33N-WGS84 Coordinate System. Morphobathymetric maps were elaborated, with a cell size of 5 × 5 m, using CARIS HIPS and SIPS and Teledyne RESON PDS2000 software. The survey area extends from 140 to 634 m depth and covered a total area of 104 km^2^.

All four ROV surveys have been conducted using a Pollux III ROV, equipped with low definition CCD video-camera for navigation and general description, and high definition (2034 × 1296 pixels) video-camera (SonyHDR-HC7) for detailed description. Three laser beams at distance of 20 cm each other, provided the scale bar on the videos. The ROV was equipped with an acoustic position system which provided the exact geographical and depth position every 1 s. Videos have been analyzed with Adelie Video and Adelie GIS based on ArcMap® Geographic Information System (distributed by ESRI). The tool “minifilms” was used to extract high-resolution still images from ROV footage every 10 seconds: 2048 photographs have been obtained with each image covering ca. 6 m^2^. A total length of 2703.36 m (more than 15,000 m^2^) was surveyed during the four dives, of which 1895.58 m pertain to mobile substrates (such as mud) and 807.77 to hard substrates (e.g., rocky outcrops).

Taxa recognition was based on still HD images analysis and on a limited number of samples obtained by the ROV’s robotic arm. Sampled organisms were first photographed, and then fixed on board in a buffered 4% formaldehyde solution or preserved in proof ethanol for genetic analyses. Taxonomic identification was conducted in the laboratory using the proper techniques of each taxonomic group, and conforms to the World Register of Marine Species^49^.

A first ROV exploration of the Dohrn Canyon habitat was conducted in June 2016 during the oceanographic cruise ANOMCITY16 onboard R/V *Minerva Uno* (station code DOHRN_ROV_1), followed by others explorations in July 2016 and July 2017 for the initial assessment and monitoring of the new site within the frame of the Italian *Marine Strategy Framework Directive* (station codes MS16_ and MS17_, respectively). In total, four ROV dives explored three distinct sectors of the canyon head’s NW branch, along transects perpendicular to the axis of the channel and ending on the steep rocky margin of the channel. Dives DOHRN_01 and MS16_7 have been the first two exploratory dives in the canyon revealing high biodiversity level (Fig. 1B,C, and Table 2). Both dives explored the northwestern flank of the canyon at depth comprised between 375 and 455 m. Deeper parts, from 420 to 455 m are characterized by gentle topography and muddy substrate with no evident mega- and macrofauna. The two dives explored in detail the canyon wall from 375 to 410 m where rocky substrate outcrop. To the west of this sector (MS17_81), the area is characterized by a relatively gentle morphology between 360–450 m water depth, with muddy sediment prevailing over scattered outcropping bedrock occurrences at 380 and 450 m respectively. The easternmost ROV survey (MS17_102) explored the canyon’s head between 345–470 m. This side of the canyon is characterized by gentle muddy topographies alternating with rocky outcrops and cliffs (vertical walls) between 440 and 460 m and 390 and 430 m respectively.

A fragment (~20 mg) of a fossil shell of *N. zibrowii* was carefully cleaned using a small diamond blade to remove any visible contamination, leached with diluted HCl and H_3_PO_4_ and analyzed for AMS-^14^C at the Poznan Radiocarbon Laboratory in Poland. The radiocarbon age was converted into calendar years (cal. yr BP, BP = AD 1950) using the Marine13^50^ calibration curve and the Calib7.0.4 program^51^. Prior to calibration, the age was corrected for an extra 49 ± 42 years regional reservoir effect (ΔR) using the value reported in the Marine Reservoir Correction Database (http://calib.qub.ac.uk/marine/).
